# Supplementary material for: Selective disruption of microtubule formation at the nuclear envelope impairs the bone resorption capacity of osteoclasts
Source: J Cell Sci. 2026 Jan 23;139(12):jcs264166. doi: 10.1242/jcs.264166 (PMC12863296; doi:10.1242/jcs.264166)
Supplement: Supplementary information [file joces-139-264166-s1.pdf]

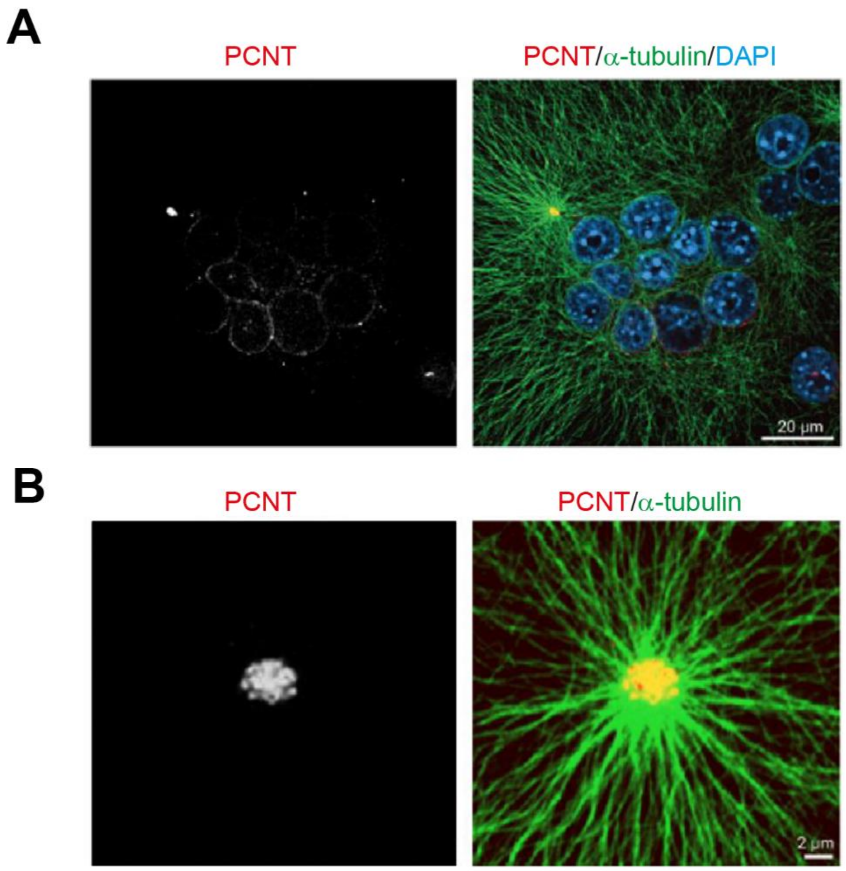

Fig. S1. Centrosome clusters in RAW264.7 derived osteoclasts. (A) Immunostaining of PCNT (red), α-tubulin (green), and DNA (DAPI) in RAW264.7-derived osteoclasts. Scale bar: 20 µm. (B) Close-up of the centrosome cluster. Scale bar: 2 µm.

Journal of Cell Science • Supplementary information

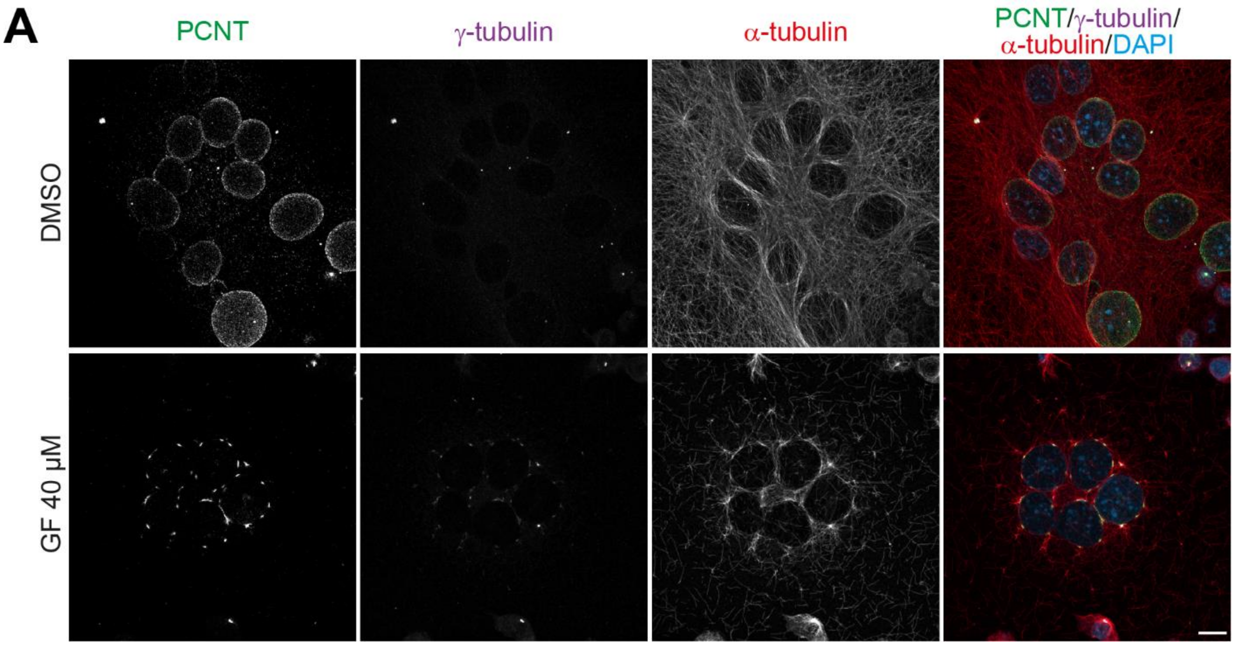

Fig. S2. Griseofulvin affects microtubule and NE-MTOC organization. (A) Immunostaining of PCNT (green), γ-tubulin (magenta), α-tubulin (red), and DNA (DAPI) in RAW264.7-derived osteoclasts treated with DMSO or 40 µM Griseofulvin (GF) for 15 h. Scale bar: 10 µm.
